# Tailoring Properties Through Functionalized Alicyclic Diamine Towards Solution-Processable High-Performance Polyimide Films

**DOI:** 10.3390/polym18020207

**Published:** 2026-01-12

**Authors:** Lei Xiong, Feiyan Ding, Liangrong Li, Xinhai Wei, Jiayao Xu, Guanfa Xiao, Zhenyu Yang, Feng Liu

**Affiliations:** 1 School of Pharmacy, Nanchang Medical College, Nanchang 330052, China; xionglei@ncmc.edu.cn (L.X.); dingfeiyan@ahnu.edu.cn (F.D.); kakate@ncmc.edu.cn (X.W.); xiaofeng@ncmc.edu.cn (J.X.); xiaoguanfa@ncmc.edu.cn (G.X.); 2 School of Chemistry and Chemical Engineering, Nanchang University, Nanchang 330031, China; 3 School of Chemistry and Materials Science, Anhui Normal University, Wuhu 241002, China; 4 Department of Pharmacy, Fuzhou Medical College, Fuzhou 344000, China; liangrongli123@163.com

**Keywords:** alicyclic diamine, solution-processable, high-performance films, hydrophobicity, structure–property relationship

## Abstract

A novel fluorinated diamine monomer, 4,4′-((bicyclo[2.2.1]hept- 5-ene-2,3-diylbis (methylene)) bis(oxy))bis(3- (trifluoromethyl) aniline) (NFDA), featuring a tailored alicyclic norbornane core, flexible ether linkages, and pendant trifluoromethyl groups, was successfully synthesized. This monomer was polymerized with six commercial dianhydrides to produce a series of poly(amic acid) precursors, which were subsequently converted into high-performance polyimide (PI) films via a thermal imidization process. The strategic integration of the alicyclic, ether, and fluorinated motifs within the polymer backbone resulted in materials with an exceptional combination of properties. These PI films display outstanding solubility in a wide range of organic solvents, including low-boiling options like chloroform and tetrahydrofuran, highlighting their superior solution processability. The films are amorphous and exhibit remarkable hydrophobicity, evidenced by high water contact angles (up to 109.4°) and minimal water absorption (as low as 0.26%). Furthermore, they possess excellent optical transparency, with a maximum transmittance of 86.7% in the visible region. The materials also maintain robust thermal stability, with 5% mass loss temperatures exceeding 416 °C, and offer a desirable balance of mechanical strength and flexibility. This unique set of attributes, stemming from a rational molecular design, positions these polyimides as highly promising candidates for next-generation flexible electronics and advanced photovoltaics.

## 1. Introduction

Polyimide (PI) is a class of aromatic heterocyclic polymers characterized by the presence of imide structures within the main chain. It exhibits outstanding thermal stability, chemical resistance, mechanical strength, and dielectric properties [1,2,3] and has found extensive applications in diverse fields such as electrical insulation, microelectronics, optoelectronics, auto-material, the aerospace industry, and electrocommunication [4,5,6]. However, due to the continuously growing demand in fields such as photovoltaic power, flexible displays, and electrocommunication, traditional aromatic PIs, which exhibit high molecular chain rigidity and are prone to the formation of intramolecular and intermolecular charge transfer complexes (CTC), are encountering challenges including poor solubility and melt processability, compromised optical transparency, elevated dielectric constants, high coefficient of thermal expansion, and proneness to moisture absorption [7,8]. Therefore, improving the solubility and transparency of PI while simultaneously reducing its water absorption and dielectric constant remains a primary challenge in current research.

To overcome these limitations, several strategies have been developed. (1) Fluorinated groups are introduced to suppress the formation of charge transfer complexes (CTC), which enhances solubility, optical transparency, and dielectric properties [9,10]. Zhang’s [11] work demonstrated that the introduction of trifluoromethyl can significantly improve the hydrophobicity of PI. The PI prepared from a new type of diamine monomer containing difluoromethyl has excellent thermal performance, optical performance, and hydrophobicity (contact angle > 91.8°) [12]. (2) Bulky alicyclic structures are introduced to impart greater chain flexibility compared to aromatic analogues and disrupt molecular conjugation and CTC formation, thereby enhancing solvent affinity [13,14]. Yu [13] prepared two types of high-temperature-resistant and low dielectric loss alicyclic PIs and applied them in the field of high-temperature energy storage capacitors. Guo [15] developed an aromatic PI containing an alicyclic structure, which exhibited uniform pore size distribution, high solvent affinity, and excellent gas separation performance. (3) Asymmetric or non-coplanar structures are employed to effectively reduce crystallinity and suppress CTC, resulting in a lower dielectric constant alongside improved solubility and transparency [16,17]. Sidra [18] prepared soluble and fusible PI films using two asymmetric diamines as monomers. Shao [19] prepared a series of asymmetric PI films using 1,4-(2′,4″-diaminodiphenoxy)benzene with commercial dianhydride, all of which exhibited high transparency. (4) Silane or siloxane segments are introduced to provide chain flexibility, inhibit chain entanglement, and improve solubility while simultaneously reducing water absorption and dielectric constant without compromising mechanical strength or thermal stability [20]. Qu [21] prepared a series of silicon-containing PIs via copolymerization of ODA, APDS, and 6FDA, with a water contact angle of 91.8°, dielectric constant below 3.5, and dielectric loss under 0.01 at high frequency. (5) Bulky side chains are grafted to increase intermolecular spacing and decrease chain packing density, which significantly improves solubility and optical transparency [12]. Zhang [22] synthesized a novel diamine monomer with tetrafluorostyrol as the side chain, which prepared PI films with not only excellent solubility and transparency but also a low dielectric constant. (6) Flexible linkages, such as ether or ester groups, are integrated to reduce chain rigidity and enhance the solubility of PI [23]. Zhang [24] studied the effect of ester on the water absorption, dielectric properties, thermal stability, and mechanical strength of PI, and the results indicated that the strong molecular interaction force caused by ester can effectively reduce the water absorption of PI, which is an ideal material for electrocommunication.

The synthesis of a conventional Kapton film typically involves the reaction of PMDA and ODA. Although the resulting structure, featuring a single flexible ether bond, imparts PI with excellent thermal stability and electrical insulation properties, it exhibits limitations in optical transparency and certain other performance aspects. The stability and optical performance of a PI film with the introduction of an alicyclic structure are superior to those with hydrophobicity [25]. In addition, although fluorine-containing polyimides exhibit notable advantages in terms of optical transparency, chemical resistance, and hydrophobicity, their high fluorine content may lead to issues such as mechanical degradation [26]. Compared with the common alicyclic structure, the non-planar norbornene has both semi-rigid and weakly conjugated structures, which gives the polymer high thermal stability and a high energy band gap and improves solubility, dielectric properties, and optical performance while maintaining the thermal stability of the PI [27]; the ether bond is more stable and flexible compared to the disadvantage of the ester group that is prone to hydrolysis. Currently, most studies have focused on the introduction of a single structure, such as fluorinated groups, alicyclic structure, or flexible groups, into the PI backbone chain [28,29,30]. In this work, trifluoromethyl, an ether bond, and a norbornene alicyclic structure were simultaneously introduced into a diamine monomer and combined with commercial dianhydrides, such as 6FDA, BPADA, ODPA, PMDA, BTDA, and BPDA, to prepare six kinds of high-performance PI films; their thermal stability, optical performance, solubility, mechanical strength, hydrophobicity, etc., were characterized; and the relationship between the structure and properties of PI was systematically investigated.

## 2. Materials and Methods

5-Norbornene-2,3-dicarboxylic Anhydride(NDA), Lithium Aluminum Hydride(LAH), 10% Palladium on Carbon(10% Pd), 2-Fluoro-5-nitrobenzotrifluoride, and sodium hydride(NaH) were purchased from Shanghai Aladdin Biochemical Technology Co., Ltd., Shanghai, China. Tetrahydrofuran (THF), N, N-Dimethylformamide (DMF), Anhydrous N, N-dimethylacetamide (DMAc), toluene(TL), 80% hydrazine hydrate, trichloromethane(CHCI_3_), dimethyl sulfoxide (DMSO), absolute ethyl alcohol(ET), ethyl acetate(EA), 4,4′-(hexafluoroisopropylidene)diphthalic anhydride (6FDA), 4,4′-Bisphenol A dianhydride(BPADA), 4,4′-Oxydiphthalic anhydride (ODPA), pyromellitic dianhydride (PMDA), 3,3′,4,4′-Benzophenonetetracarboxylic dianhydride(BTDA), and 3,3′,4,4′-Biphenyltetracarboxylic dianhydride(BPDA) were purchased from Sinopharm Chemical Reagent Co., Ltd., Shanghai, China.

FT-IR spectra (KBr) were recorded on an FTS-40 Fourier transform infrared spectrometer, Thermo Fisher Scientific, Massachusetts, USA, with a scanning range of 400~4000 cm^−1^. ^1^H NMR and ^13^C NMR spectra were measured on a DRX500 spectrometer, Bruker, Saarbrücken, Germany, with tetramethylsilane (TMS) as the internal standard and d-DMSO as the solvent. Thermogravimetric analysis (TGA) was conducted with a Q500, TA Instruments, State of Delaware, USA, and experiments were carried out on approximately 10 mg of samples in flowing nitrogen at a heating rate of 20 °C/min and test temperature range 40~800 °C. Differential scanning calorimetry (DSC) testing was performed on a DSC 214 Polyma, Netzsch, Selb, Germany, at a scanning rate of 20 °C/min in flowing nitrogen and test temperature range 75~375 °C, and glass transition temperatures (T_g_) were read from the DSC curves at the same time. Gel permeation chromatography (GPC) was performed on a GPC E2695, Waters, MA, USA, with DMAC as the solvent. Wide-angle X-ray radiometry (WAXRD) was performed on a SmartLab 9KW, Rigaku, Tokyo, Japan, with a Cu/Kα reflector (λ = 0.154 nm) as the source of the test rays and a scanning range of 5 to 55 o. Contact angle measuring instrument was performed on a CA200 by Guangdong Beidou Precision Instrument Co., Ltd., Guangdong, China, with the solvent being water. UV–Vis spectrophotometry (UV–Vis) was performed on an 1800PC by Shanghai Mapada Instrument Co., Ltd., Shanghai, China, with a scanning range of 350~800 nm at room temperature. Fully automatic video melting point testing was performed on a JH80 Shanghai Precision Instrumentation Co., Ltd., Shanghai, China, Tensile testing was performed on a TST-01H Intelligent Electronic Tensile Testing Machine (Jinan Zhongce Mechanical & Electrical Equipment Co., Ltd., Jinan, China.) at a constant displacement rate of 200 mm/min. The optimized structures and orbital energies of various types of PI simulated compounds were obtained by density-functional theory (DFT) calculations using the software version Gaussian 16 A.03.

## 3. Results

### 3.1. Synthesis of the Diamine Monomer

**Scheme 1:**
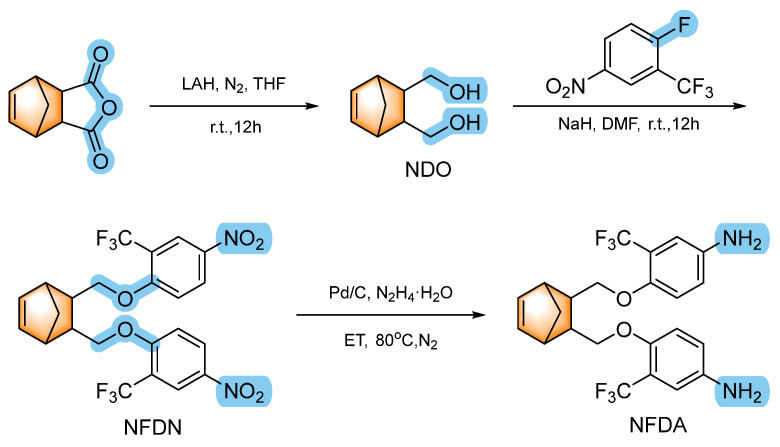
Synthesis route of NFDA.

The synthesis of 4,4′-((bicyclo[2.2.1]hept-5-ene-2,3-diylbis(methylene)) bis(oxy)) bis(3-(trifluoromethyl)aniline) (NFDA) was carried out in three steps as shown in Scheme 1.

#### 3.1.1. Synthesis of Bicyclo[2.2.1]hept-5-ene-2,3-diyldimethanol (NDO)

N_2_ atmosphere, LAH (9.0760 g, 0.2391 mol), and THF (60 mL) were added to a 500 mL three-necked flask in an ice bath. Thereafter, 60 mL of THF and 16.4160 g (0.1000 mol) of a mixture of NDA were slowly dripped into the system and stirred for 12 h at room temperature. The unreacted LAH was quenched by adding an appropriate amount of distilled water dropwise and filtered; the filtrate was concentrated until a large amount of white solid was precipitated, it was filtered, and the residue was recrystallized with THF and dried in a vacuum at 55 °C for 5 h to obtain 7.8426 g of white crystals of NDO, with the yield: 50.86%, m.p.: 85.1~85.7 °C. FT-IR, σ/cm^−1^: 3261(O-H), 1648(C=C); ^1^H-NMR (400 MHz, DMSO), δ: 6.03(s, 2H), 4.41(s, 2H), 3.18(J = 4.00 Hz, d, 4H), 2.79(s, 2H), 2.25–2.18(m, 2H), 1.27(J = 8.00 Hz, d, 2H); ^13^C-NMR(DMSO), δ: 135.36, 61.34, 49.01, 45.53, 44.60, 40.57, 40.36, 40.15, 39.94, 39.73, 39.52, 39.31.

#### 3.1.2. Synthesis of 5,6-Bis((4-nitro-2-(trifluoromethyl)phenoxy)methyl)bicyclo[2.2.1] hept-2-ene (NFDN)

A total of 1.9800 g NaH was added into a 250 mL three-necked flask along with N_2_ atmosphere; then, slowly add the mixture of NDO (2.3132 g, 0.015 mol) dissolved in 15 mL of DMF, followed by a 60 °C reaction for 3 h, which was then reduced to room temperature. 2-Fluoro-5-nitrobenzotrifluoride (7.3185 g, 0.0350 mol) was added dropwise, the system became brownish-yellow, and the reaction was 12 h at room temperature. We then slowly added distilled water dropwise until a large number of solids precipitated, which were then extracted. The filtrate was recrystallized with DMF and dried in a vacuum at 100 °C for 10 h to obtain 2.5390 g of yellow solid NFDN, with the yield: 31.79%, m.p.: 149.3~150.3 °C. FT-IR, σ/cm^−1^: 1621(C=C), 1525(N=O), 1340(N=O), 1170(C-O), 1141(C-O), 900(C-N); ^1^H-NMR(400 MHz, DMSO), δ: 8.49(J = 8.20, 2.40 Hz, dd, 2H), 8.35(J = 2.40 Hz, d, 2H), 7.47(J = 9.20 Hz, d, 2H), 6.16(s, 2H), 4.22(J = 9.20, 4.40 Hz, dd, 4H), 3.00(s, 2H), 2.91–2.46(m, 2H), 1.45(s, 2H); ^13^C-NMR(DMSO), δ: 161.56, 140.43, 135.92, 130.47, 123.39, 121.52, 117.75, 114.79, 70.50, 48.64, 45.20, 40.39, 40.27, 39.95, 39.74, 39.53, 39.32.

#### 3.1.3. Synthesis of 4,4′-((Bicyclo[2.2.1]hept-5-ene-2,3-diylbis(methylene))bis(oxy)) bis(3-(trifluoromethyl)aniline) (NFDA)

Pd/C (0.1300 g), NFDN (2.6620 g, 0.0050 mol), and ET (60 mL) were added to a 250 mL three-necked flask, which was heated to 50 °C under N_2_ atmosphere, and then 10 mL of 80% hydrazine hydrate was added dropwise, and the system was refluxed at 80 °C for 12 h until it turned black. It was filtered three times until no black solid was present on the filter paper, concentrated to a light yellow transparent filtrate, and then underwent ice bath crystallization, filtration, column chromatography purification of the product (PE: EA = 1:1), and drying in a vacuum at 35 °C for 10 h to obtain 1.4469 g of light yellow crystals NFDA, with the yield: 61.25%, m.p.: 112.3~113.3 °C. FT-IR, σ/cm^−1^: 3431(N-H), 3340(N-H), 1678(C=C); ^1^H-NMR(400 MHz, DMSO), δ: 6.93(J = 8.80 Hz, d, 2H), 6.73–6.72(m, 2H), 6.57(s, 2H), 5.01(s, 2H), 4.07–3.87(m, 4H), 2.46–2.30(m, 2H), 1.45–1.31(m, 2H), 1.27–1.05(m, 2H); ^13^C-NMR(DMSO), δ: 147.56, 142.66, 125.76, 123.06, 118.83, 117.89, 115.62, 111.93, 67.06, 40.57, 40.36, 40.06, 39.42, 22.26.

### 3.2. The Preparation of PI Films

**Scheme 2:**
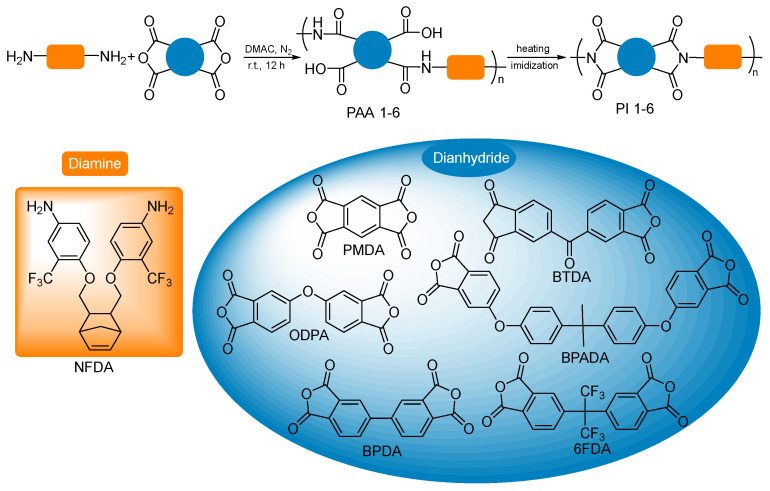
Synthesis route of PI 1~6.

The one-step polycondensation of NFDA with six commercial dianhydrides for the preparation of PI is shown in Scheme 2, and the preparation of PI 1 from NFDA and 6FDA, for example, is shown below. We added 1.8897 g (0.004 mol) NFDA and 1.7770 g (0.004 mol) 6FDA into a 50 mL three-necked flask, with anhydrous DMAC as solvent and 30% solid content, and stirred at room temperature under the protection of N_2_ for 12 h to obtain viscous and transparent polyamidoacetic acid solution (PAA 1). The film was made by the calendering method on a glass plate, and the film was prepared according to the following conditions: 80 °C (5.5 h), 100 °C (1.5 h), 150 °C (1.5 h), 200 °C (1.5 h), 250 °C (1.5 h) of the process will be PAA 1 thermal imidization, the glass plate with PI was immersed in distilled water and then peeled to obtain the thickness of 83 μm polyimide film PI 1 (6FDA-NFDA). The thickness of the films used for optical performance testing is 20 to 25 μm, and the mechanical strength and other tests are 70 to 90 μm. PI 2 (BPADA-NFDA), PI 3 (ODPA-NFDA), PI 4 (PMDA-NFDA), PI 5 (BTDA-NFDA), and PI 6 (BPDA-NFDA) were prepared using the same method described above.

PI 1(NFDA-6FDA). FT-IR, σ/cm^−1^: 2960, 2883, 1786(C=O), 1734(C=O), 1623(C=C), 1507, 1439, 1380, 1324, 1255(C-N), 1137, 1052, 720; ^1^H-NMR(300 MHz, DMSO), δ/ppm: 8.19–8.16(m, 2H), 8.10–7.98(m, 2H), 7.83–7.74(m, 4H), 7.49(J = 1.80 Hz, d, 2H), 7.32(s, 2H), 4.33(J = 0.90 Hz, d, 1H), 4.20(J = 1.50 Hz, d, 2H), 3.77(J = 4.80 Hz, d, 2H), 2.39(s, 1H), 2.01(s, 1H), 1.52(s, 3H), 1.34(d, 1H), 1.18(J = 3.60 Hz, t, 4H).

PI 2(NFDA-BPADA). FT-IR, σ/cm^−1^: 2964, 2879, 1779(C=O), 1724(C=O), 1619(C=C), 1504, 1438, 1377, 1276(C-N), 1135, 1053, 1014, 849; ^1^H-NMR(300 MHz, DMSO), δ/ppm: 7.94–7.91(m, 2H), 7.71(J = 0.90 Hz, d, 4H), 7,45–7.33(m, 12H), 7.12(s, 2H), 4.36(J = 0.30 Hz, d, 2H), 4.13(s, 1H), 3.71(s, 2H), 2.39(s, 1H), 2.01(s, 1H), 1.70(s, 6H), 1.52(s, 1H), 1.37(J = 0.30 Hz, d, 4H).

PI 3(NFDA-ODPA). FT-IR, σ/cm^−1^: 2960, 1778(C=O), 1725(C=O), 1611(C=C), 1503, 1469, 1378, 1322, 1276(C-N), 1135, 1053, 1007, 744; ^1^H-NMR(300 MHz, DMSO), δ/ppm: 8.07(s, 2H), 7.73(J = 2.40 Hz, d, 4H), 7.62(s, 2H), 7.47(s, 2H), 7.27(J = 1.80 Hz, d, 2H), 4.33(d, 2H), 4.14(s, 1H), 3.76(J = 6.60 Hz, d, 2H), 2.40(s, 1H), 2.02(s, 1H), 1.54(s, 3H), 1.35(s, 1H), 1.18(J = 3.90 Hz, t, 4H).

PI 4(NFDA-PMDA). FT-IR, σ/cm^−1^: 2954, 2881, 1775(C=O), 1726(C=O), 1621(C=C), 1506, 1439, 1379, 1322, 1258(C-N), 1217, 1128, 1053, 738; ^1^H-NMR(300 MHz, DMSO), δ/ppm: 8.44–8.26(m, 2H), 7.79–7.68(m, 4H), 7.34–7.27(m, 2H), 4.39(J = 13.8 Hz, d, 2H), 4.21(J = 1.50 Hz, d, 1H), 3.93–3.73(m, 2H), 2.43(s, 1H), 2.02(s, 1H), 1.59(s, 3H), 1.52–1.35(m, 1H), 1.13(s, 4H).

PI 5(NFDA-BTDA). FT-IR, σ/cm^−1^: 2967, 2881, 778(C=O), 1727(C=O), 1611(C=C), 1562, 1508, 1426, 1370, 1274(C-N),1138, 1052, 1011, 899, 823, 753; ^1^H-NMR(300 MHz, DMSO), δ/ppm: 8.40–9.31(m, 2H), 7.96–7.84(m, 4H), 7.70–7.71(m, 4H), 7.33–7.22(m, 2H), 4.36–4.03(m, 2H), 4.00–3.94(m, 1H), 3.77–3.64(m, 2H), 2.60(J = 13.80Hz, d, 1H), 1.92(J = 2.70Hz, d, 1H),1.54–1.45(m, 2H), 1.29(s, 1H), 1.19(s, 1H), 1.12(s, 3H).

PI 6(NFDA-BPDA). FT-IR, σ/cm^−1^: 2952, 2880, 1776(C=O), 1726(C=O), 1621(C=C), 1507, 1439, 1380, 1332, 1258(C-N), 1217, 1123, 1053, 1007, 901, 880, 839, 736, 665; ^1^H-NMR(300 MHz, DMSO), δ/ppm: 8.08–8.00(m, 2H), 7.92(s, 4H), 7.86(J = 5.40 Hz, d, 4H), 7.28(J = 4.50 Hz, d, 2H), 4,42(s, 2H), 4.22(J = 3.00 Hz, d, 1H), 3.34(s, 2H), 2.46(s, 1H), 2.03(s, 1H), 1.59–1.35(m, 6H), 1.14(s, 2H).

### 3.3. The Characterization of NFDA

**Figure 1:**
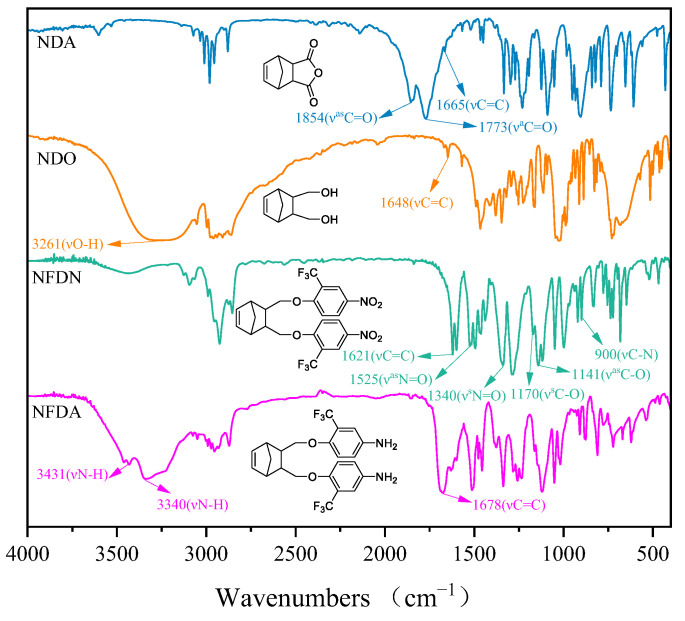
FT-IR spectra of NDA, NDO, NFDN, and NFDA.

**Figure 2:**
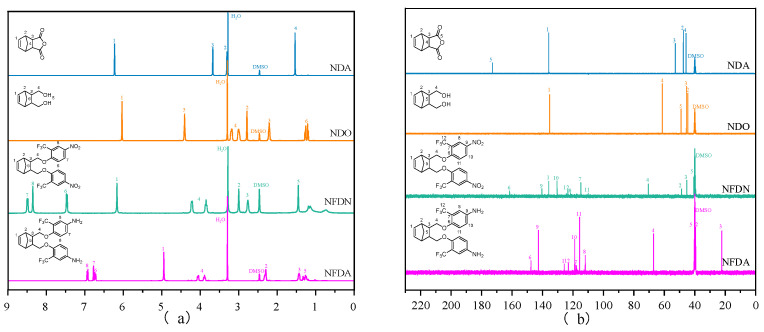
(**a**) ^1^H NMR spectra of NDA, NDO, NFDN, and NFDA; (**b**) ^13^C NMR spectra of NDA, NDO, NFDN, and NFDA.

The structure was characterized in detail using FT-IR, ^1^H NMR, and ^13^C NMR. The FT-IR, ^1^H NMR, and ^13^C NMR spectra of NFDA and its intermediates are shown in Figure 1, Figure 2a, and Figure 2b, respectively, and the signals in the spectra were assigned in detail.

Figure 1 shows that the absorption peaks at 1854 cm^−1^ and 1773 cm^−1^ in the NDA spectrum are the asymmetric stretching vibration (νas) and symmetric stretching vibration (νs) of the carbonyl group (C=O). The 1665 cm^−1^ peak corresponds to the stretching vibration of the carbon–carbon double bond (C=C) in the ring. The appearance of the hydroxyl (-OH) stretching vibration absorption peak at 3261 cm^−1^ and the disappearance of the characteristic absorption peak of the carbonyl (C=O) in the NDO spectrum both indicate that the acid anhydride in the NDA has been reduced to a hydroxyl group. The νas and vs of the ether bond (C-O-C) appearing at 1141 cm^−1^ and 1170 cm^−1^ in the NFDN spectrum indicate that the F atom in 2-Fluoro-5-nitrobenzotrifluoride has been successfully replaced by NDO. The characteristic absorption peaks of amino nitrogen hydrogen (-NH) stretching vibrations appearing at 3431 cm^−1^ and 3340 cm^−1^ in the NFDA spectrum indicate that NFDN has been successfully reduced to NFDA.

As can be seen from the NFDA spectrum in Figure 2a, signal peak 5 at δ 1.29 belongs to the hydrogen on the norbornene bridge carbon; signal peak 3 (δ 1.43) belongs to the methylene hydrogen near the ether bond on the norbornene ring; signal peak 2 (δ 2.28) belongs to the methylene hydrogen close to the double bond on the norbornene ring; signal peak 4 (δ 4.05~3.89) belongs to the hydrogen on the methylene carbon connected to the ether bond; the hydrogen on the double bond carbon corresponds to signal peak 1, with a chemical shift of 5.01; and signal peaks 6, 7, and 8 correspond to the three hydrogen atoms on the benzene ring, with chemical shifts of 6.57, 6.74, and 6.93, respectively.

As can be seen from the NFDA spectrum in Figure 2b, signal peak 3 (δ 22.26) belongs to the methylene carbon near the ether bond on the norbornene ring; and signal peaks 2 and 5 (δ 39.42~40.57) belong to the two bridge carbons on norbornene. The signal peak at δ 67.06 belongs to the methylene carbon connected to the ether bond. Due to its connection with oxygen, the chemical shift moves to a lower field. Signal peaks 6 (δ 147.56), 7 (δ 117.89), 8 (δ 111.93), 9 (δ 142.66), 10 (δ 118.83), and 11 (δ 115.62) are attributed to the carbon atoms on the benzene ring; the signal peak at δ 125.76 is attributed to the carbon atom of the double bond; and the signal peak at δ 123.06 is attributed to the carbon atom of the trifluoromethyl group.

### 3.4. The Structural Characterization of PI

**Figure 3:**
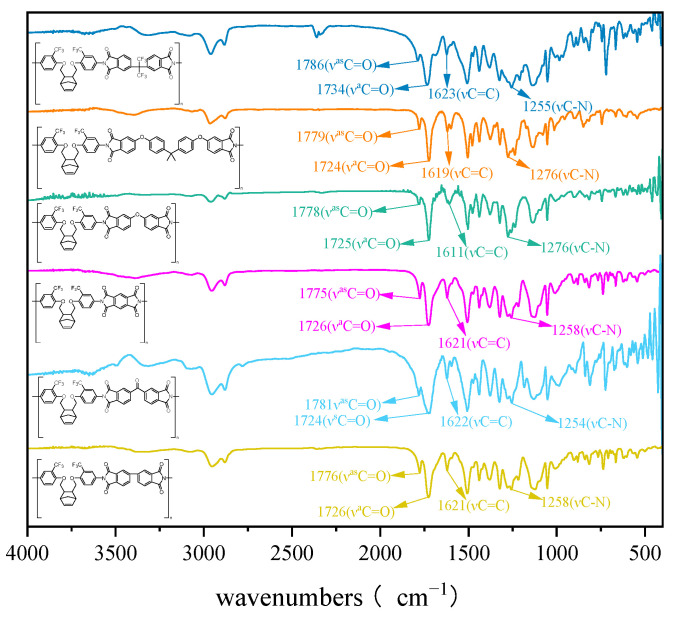
FT-IR spectra of PI 1~6.

**Figure 4:**
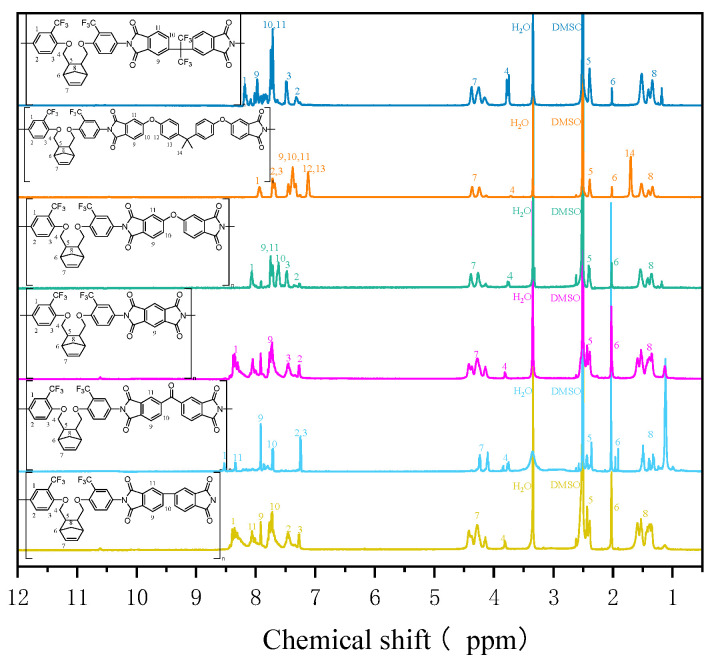
^1^H-NMR spectra of PI 1~6.

The structure and molecular weight of PI were characterized by FT-IR, ^1^H NMR, and GPC, and the results are shown in Figure 3, Figure 4, and Table 1, respectively.

As can be seen from Figure 3, the absorption peaks near 1775 cm^−1^ and 1726 cm^−1^ are the asymmetric and symmetric stretching vibrations of the carbonyl group (C=O) of the imide ring, respectively, and the absorption peak near 1274 cm^−1^ is the stretching vibration of the carbon–nitrogen bond (C-N) of the imide ring. In addition, no stretching vibrational peaks of amino nitrogen–hydrogen bonding (N-H) appeared at 3300~3500 cm^−1^, indicating a high degree of PI polymerization. In Figure 4, the signal peaks at δ 7.25~8.35 are attributed to the hydrogen on the benzene ring, the signal peaks at δ 3.71~4.39 are attributed to the hydrogen on the ether bond adjacent to the methylene group and carbon–carbon double bond, and the signal peaks at δ 1.12~2.46 are attributed to the hydrogen on the methylene group and the methine group, and the results are in agreement with the molecular structure of the designed PI molecule.

As shown in Table 1, the number average molecular weight (Mn) of PI ranged from 32.6 to 55.8 × 10^4^, and the weight average molecular weight (Mw) ranged from 39.7 to 59.9 × 10^4^; the molecular weight distribution indices (Mw/Mn) ranged from 1.07 to 1.21, which indicated that PI 1~6 had been successfully polymerized.

### 3.5. Solubility in Organic Solvent

The solubility results of PI 1~6 are shown in Table 2. The series of PI not only has excellent solubility in high-boiling solvents, such as DMF, DMSO, DMAC, etc., but also has good performance in low-boiling solvents, such as THF, EA, CHCI_3_, etc., which has greatly broadened the processing window of PI. This is mainly attributed to the introduction of the norbornene structure and trifluoromethyl. The non-coplanar structure of norbornene increases the intermolecular distance between PI molecules [31], reduces intermolecular van der Waals forces and the degree of close packing, and alleviates the conjugation of molecular chains, making it difficult for CTC and crystals to form, which is conducive to improving PI solubility. The trifluoromethyl group, which has a strong electron-withdrawing effect, not only breaks the electron conjugation of the molecular chain but also increases the PI chain spacing, free volume fraction (FFV), and molecular flexibility, disrupting the regularity of the molecule and allowing solvent molecules to diffuse more easily into the PI interior, thereby improving its solubility.

**Figure 5:**
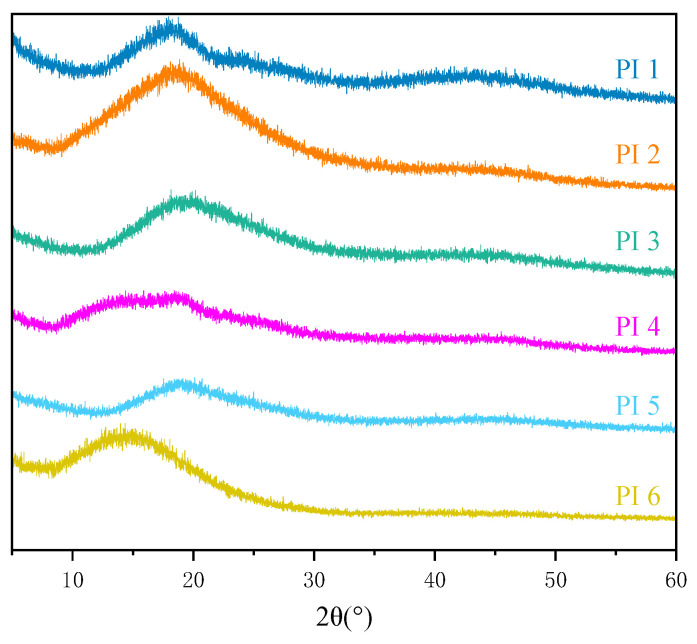
WAXRD patterns of PI 1~6.

The WAXRD spectra of PI 1~6 are shown in Figure 5. The WAXRD curves of the six PI all exhibit broad diffuse peaks, indicating that they all have amorphous structures. Based on the highest points of the PI 1~6 diffraction peaks in Figure 5, the average interchain spacing was calculated using Bragg’s Equation (1). Table 3 lists the average interchain distances for PI 1~6, which are 6.81, 6.37, 6.70, 4.78, 5.48, and 5.89 Å, respectively, all of which are higher than those of Kapton film [32]. The larger the average interchain distance of polymers, the lower the degree of interchain entanglement and crystallinity and the higher the solubility and transparency [33]. This shows that the norbornene structure and trifluoromethyl group are indeed effective in reducing the mutual entanglement and chain stacking density of PI molecules and have a significant effect on improving the solubility and transparency of PI. However, the differences in the average interchain spacing of the six films mainly depend on the structure of the dianhydride monomers. The dianhydride monomers of PI 1 (6FDA), PI 2 (BPADA), and PI 3 (ODPA) contain trifluoromethyl, methyl, and other highly steric groups and flexible ether bonds, resulting in a larger average interchain spacing of the polymers. PI 2 and PI 3 have slightly smaller average interchain distances, which is attributed to the fact that the ether bonds contained in the two dianhydride monomers easily form hydrogen bonds, hindering the movement of the molecular chains and bringing the interchain distances closer together. PI 4 (PMDA), PI 5 (BTDA), and PI 6 (BPDA) have strong conjugated effects of dianhydride molecules and high molecular chain stacking density, and PI 4 has the greatest rigidity, resulting in the smallest chain spacing. The ketone carbonyl groups contained in PI 5 dianhydride monomers can form hydrogen bonds, resulting in more compact molecular chain stacking, and therefore, the average interchain distance is slightly lower than that of PI 6.

2dsinθ = nλ, (1)

d: average interchain distance of the polymer; θ: angle between the incident ray and the reflected ray and the reflecting crystal plane; λ: wavelength taken as 0.154; n: order of reflection taken as 1.

### 3.6. Hydrophobicity of PI

**Figure 6:**
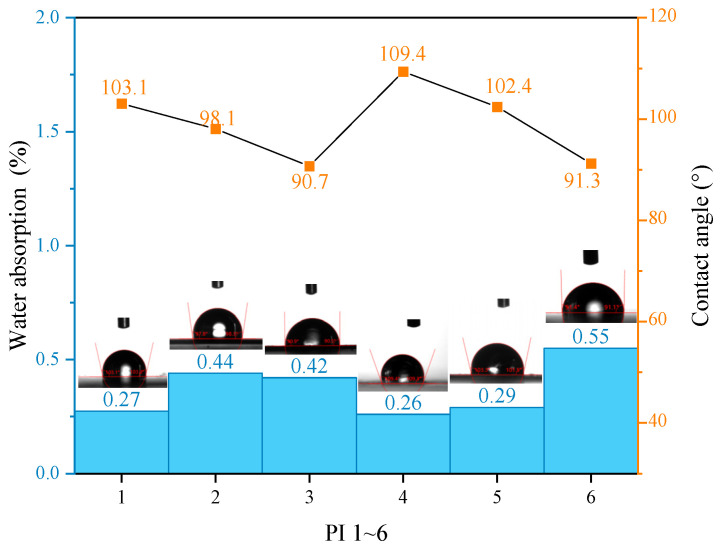
Moisture absorption rate and contact angle of PI 1~6.

PI 1~6 were cut into 3 × 3 cm samples, weighed, and immersed in distilled water at 25 °C. After 48 h, they were removed, dried, and weighed again. The water absorption rates of PI 1~6 were calculated using Equation 2. The experiment was repeated five times, and the average values are shown in Figure 6. The water absorption rates of PI 1~6 are all below 0.55%, and the water absorption rate of PI 4 is only 0.26%. Figure 6 shows contact angle test photos for PI 1 to 6, with the relevant values shown in Table 3. Except for PI 3 and PI 6, whose contact angles are slightly greater than 90°, PI 1, PI 2, PI 4, and PI 5 have contact angles of 103.1°, 98.1°, 109.3°, and 102.4°, respectively, all exhibiting excellent hydrophobicity. This is because the carbonyl oxygen and nitrogen in the traditional aromatic PI main chain imide ring have high polarizability and strong electronegativity, which can form stable hydrogen bonds with water molecules [34], and the ether bonds and ester groups commonly found in the main chain also exacerbate the tendency to form hydrogen bonds with water [35], causing the PI surface to easily form a hydrophilic interface. However, the fluorine atom in trifluoromethyl has high electronegativity, low polarizability, and high C-F bond energy, making it difficult to form hydrogen bonds with water molecules as a hydrogen bond donor [36], which greatly reduces the erosion of PI by water molecules. On the other hand, norbornene has a certain degree of rigidity and can form local microcrystals within the molecule. The presence of microcrystals hinders the penetration and diffusion of water molecules within the polymer [37], which is one of the reasons why PI 1~6 have excellent hydrophobicity. Due to the differing rigidity of the dianhydride monomers constituting PI 1~6, the number of local microcrystals within the molecules varies, resulting in differences in hydrophobicity. The results align with the order of molecular rigidity among the six dianhydride molecules: PI 4 (PMDA) > PI 1 (6FDA) > PI 5 (BTDA) > PI 6 (BPDA) > PI 3 (ODPA) > PI 2 (BPADA). The larger contact angle observed for PI 2 (BPADA) compared to PI 6 (BPDA) may be attributed to the presence of additional ether bonds in the BPADA structure. The introduction of a significant number of flexible chain segments enhances the mobility of the molecular chains, thereby facilitating the formation of π–π stacking and microcrystalline structures in the macromolecular conformation [38]. The resulting local microcrystals effectively hinder the diffusion of water molecules and increase hydrophobicity.

(2) $$w=\frac{m_{2}-m_{1}}{m_{1}}$$

w: water absorption rate; m_1_: film mass before immersion; m_2_: film mass after immersion.

### 3.7. Thermal Properties of PI

**Figure 7:**
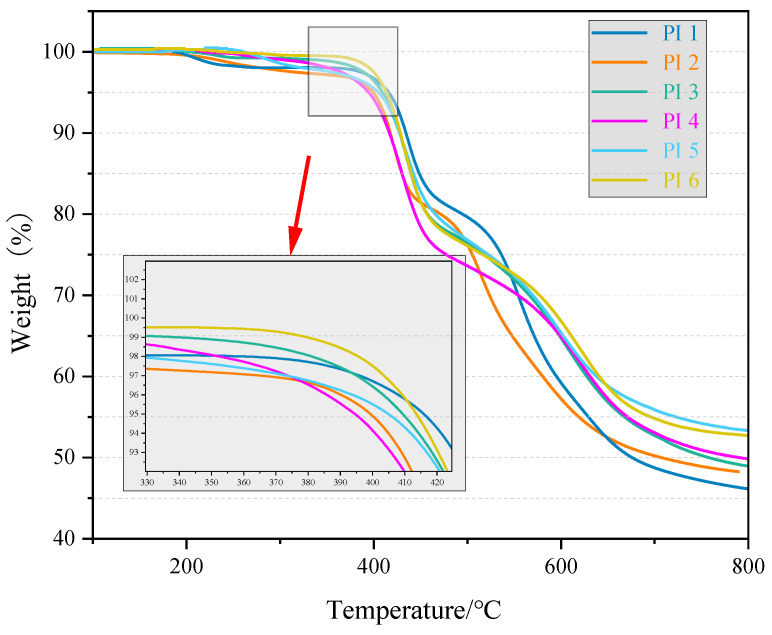
TGA curves of PI 1~6.

**Figure 8:**
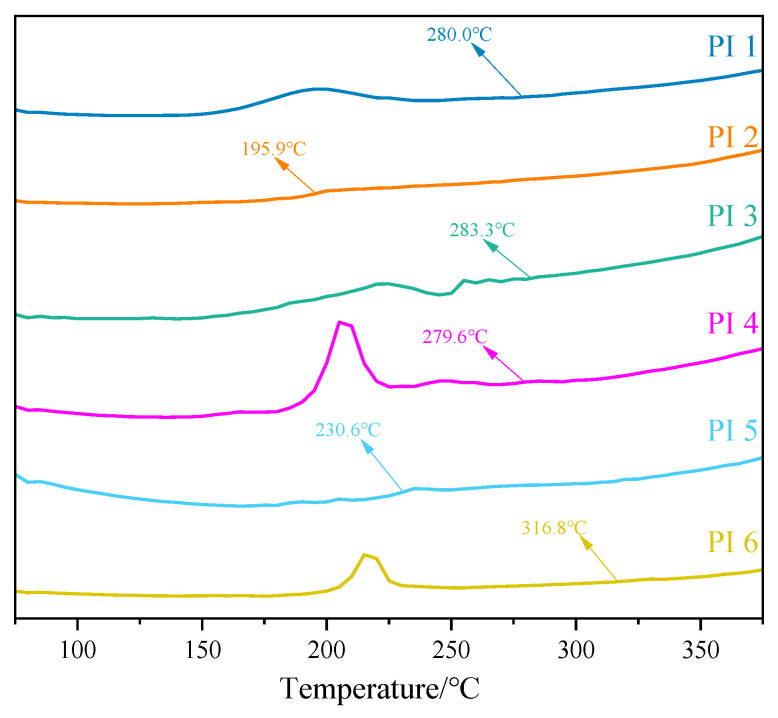
DSC curves of PI 1~6.

Figure 7 and Figure 8 show the TGA and DSC curves for PI 1~6, with the relevant values shown in Table 1. As shown in Figure 7, in a N_2_ atmosphere, PI 6 shows no significant thermal weight loss before 400 °C. The slight thermal weight loss observed in PI 1~5 may be attributed to residual water and solvents in the film. The 10% thermal weight loss temperature (T_10_) of PI 1~6 is generally above 420 °C, and 50% thermal weight loss occurs at around 800 °C, indicating that all six types of PI have good thermal stability. On the one hand, the presence of rigid groups such as benzene rings in the molecular chain restricts the movement of the molecular chain; on the other hand, the semi-rigid structure of norbornene also provides strong support for the thermal stability of PI. The combination of the two results in excellent thermal stability for the six types of PI. Above 500 °C, the thermal weight loss of PI 1 is significantly higher than that of PI 2~6. This may be due to the higher content of trifluoromethyl groups in PI 1, which significantly reduces π−π stacking interactions, resulting in more pronounced thermal weight loss above 500 °C [39].

The Tg values of PI 1~6 are shown in Table 1. The results indicate that the Tg values of all six films are between 195.9 and 316.8 °C. Notably, distinct endothermic peaks prior to Tg were observed in the DSC curves for PI 4 and PI 6, while such peaks were absent in PI 1, PI 2, PI 3, and PI 5. This phenomenon can be attributed to the combined effects of chain rigidity and the relaxation of internal stresses or metastable structures formed during film processing. Although the general trend of chain rigidity follows the order PI 4 > PI 1 > PI 5 > PI 6 > PI 3 > PI 2, the appearance of endothermic peaks is not solely determined by rigidity but also by the ability of the polymer chains to relax during thermal treatment. The highly rigid and symmetric structure of PI 4 leads to significant frozen-in stresses during cooling, which relax upon reheating, giving rise to an endothermic peak. For PI 6, its moderate rigidity combined with the semi-rigid biphenyl structure may result in specific chain packing or localized metastable states that relax in a detectable thermal event. In contrast, PI 1, despite its high rigidity, contains bulky -C(CF_3_)_2_- groups that increase free volume and enhance local chain mobility, facilitating stress dissipation during processing and thus suppressing the endothermic response. Similarly, the more flexible and asymmetric structures of PI 5, PI 3, and PI 2 allow for greater chain relaxation during film formation, minimizing residual stresses or metastable configurations and resulting in no discernible thermal peaks before Tg.

### 3.8. Optical Performance of PI

**Figure 9:**
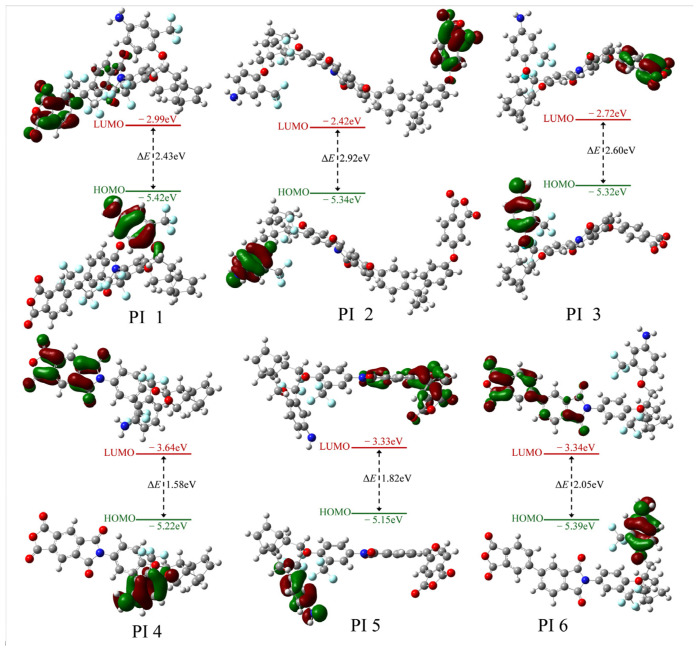
Molecular orbital energies simulation of PI 1~6 by DFT.

**Figure 10:**
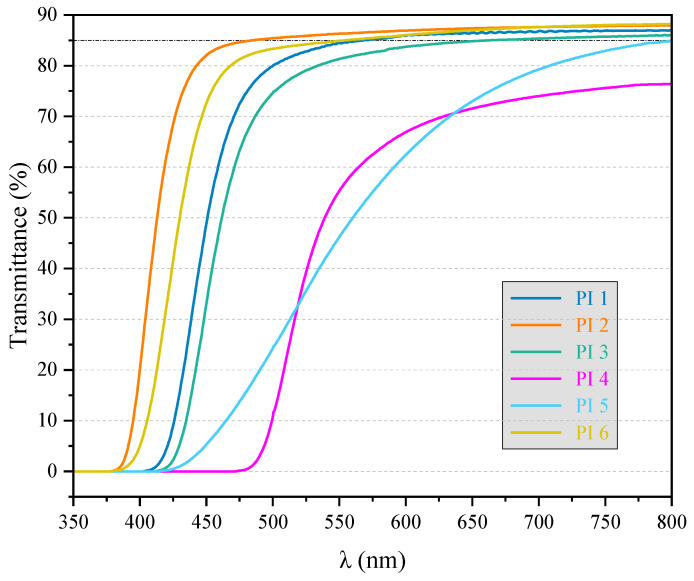
UV–Vis spectra of PI 1~6.

Taking a repetitive unit, using chemdraw3D modeling, and using Gaussian 16W for structure optimization and calculation, based on density functional theory (DFT) calculations using the 6–31G(d, p) basis set, the influence of molecular orbital energy levels on the optical properties of the polymer was investigated, yielding the values for the lowest occupied molecular orbital (E_LUMO_), highest occupied molecular orbital (E_HOMO_), and molecular orbital energy gap (ΔE = E_LUMO_-E_HOMO_), as shown in Table 3 and Figure 9. The absolute values of E_LUMO_ and E_HOMO_ are, respectively, negatively correlated with the electrophilicity of anhydrides and positively correlated with the nucleophilicity of amines. The absolute value of ΔE is negatively correlated with the formation of CTC. The magnitude of ΔE can be used to predict and assess the optical transparency of the PI polymer intuitively. The transmittance (T) and cutoff wavelength (λc) of PI 1~6 with thicknesses ranging from 72 to 85 μm were measured using UV–Vis spectroscopy, as shown in Figure 10 and Table 3. In the range of 475 to 800 nm, the transmittance of PI 1, PI 2, PI 3, and PI 6 is basically greater than 85%; it outperforms commercial PI films, such as Kapton [40], in terms of optical performance. This can be attributed to the molecular flexibility brought about by the ether bonds in the new diamine and the increased FFV between polymer chains due to trifluoromethyl, both of which can effectively curb the CTC effect within and between molecular chains and improve the transparency of PI films. The differences in optical performance between PI 1 and PI 6 are mainly related to the structure of the dianhydride monomer. The trifluoromethyl group in PI 1 dianhydride increases the interchain FFV and reduces the nucleophilicity of diamine, effectively compensating for the relatively high rigidity of 6FDA. The large number of ether bonds in PI 2 and PI 3 dianhydrides increases the flexibility of the molecular chain, and PI 6 dianhydride has relatively low rigidity, resulting in excellent optical performance for all four films. The rigidity of PMDA in PI 4 is the highest among the six dianhydrides, which most strongly inhibits molecular chain movement and leads to the most uneven electron density distribution. This structural feature promotes intermolecular charge transfer (CTC). Meanwhile, the carbonyl group of BTDA in PI 5 serves as an electron-rich donor, also facilitating the formation of strong intermolecular CTC bonds. According to DFT simulations in Figure 9, both PI 4 and PI 5 exhibit the smallest ΔE values. A smaller ΔE indicates a higher tendency for CTC formation, which is known to cause yellowing and thus results in the poorer optical performance observed in both compounds.

### 3.9. Mechanical Strength of PI

The data for tensile strength, modulus, and elongation at break are summarized in Table 1. The tensile strength ranges from 68.5 to 88.4 MPa, the tensile modulus is between 0.80 × 10^3^ and 1.60 × 10^3^ MPa, and the elongation at break is from 6.21% to 10.26%. The tensile strength and tensile modulus of PI 2 and PI 3 are slightly lower than those of the others because the large number of ether bonds in the dianhydrides of PI 2 and PI 3 enhances the flexibility of the molecular chains.

## 4. Discussion

Based on molecular structure analysis, the conjugation strengths of the six polymers can decrease in the following order: PI 5 ≈ PI 6 > PI 2 > PI 3 > PI 4 > PI 1. Stronger conjugation effects are usually accompanied by more extensive electron delocalization, which helps to enhance the thermal stability of materials but often has adverse effects on optical transparency and solubility. As shown in Table 1, PI 5 and PI 6, with the strongest conjugated structure, exhibit the highest thermal stability, and their glass transition temperatures (Tg) are approximately 25 °C higher than those of the PI 1 to PI 4 systems. This result directly confirms the contribution of conjugated structures to thermal stability. However, the enhancement of the conjugate effect also brings about obvious performance trade-offs. In terms of solubility (Table 2), PI 1 to PI 3, with weak conjugation, performed well in all test solvents, while the solubility of PI 4 to PI 6, with strong conjugation, decreased significantly, among which PI 5 and PI 6 were the most difficult to dissolve. In terms of optical performance (Table 3), the films of PI 1 to PI 3 show high light transmittance in the visible light region, while the light transmittance of PI 4 to PI 5 is significantly reduced.

## 5. Conclusions

In this study, a series of polyimide films were successfully synthesized from a novel fluorinated diamine monomer, NFDA, incorporating alicyclic norbornene, flexible ether, and trifluoromethyl motifs in combination with various commercial dianhydrides. The resulting polymers exhibit an outstanding combination of properties, driven by this rational molecular design.

These PIs display excellent solubility not only in polar aprotic solvents but also in common low-boiling solvents, such as chloroform and tetrahydrofuran, indicating superior solution processability. They also possess remarkable thermal stability, with 10% mass loss temperatures exceeding 420 °C and glass transition temperatures up to 230.9 °C. Optically, the films are highly transparent, with a maximum transmittance of 86.7% and band gaps modulated between 1.58 and 2.92 eV. Mechanically, they demonstrate robust performance, with tensile strength up to 88.4 MPa. Furthermore, the films exhibit excellent hydrophobicity, evidenced by water contact angles as high as 109.4° and 48 h water absorption rates as low as 0.26%, significantly outperforming many conventional polyimides.

In summary, the synergistic incorporation of the trifluoromethyl group, flexible ether linkages, and the semi-rigid norbornane structure effectively disrupts chain packing and suppresses charge transfer complexes. This strategy simultaneously enhances solubility, optical transparency, and hydrophobicity while maintaining the essential thermal and mechanical properties inherent to polyimides. This unique portfolio of characteristics positions these solution-processable, high-performance polyimide films as highly promising candidate materials for next-generation applications in flexible electronics, high-frequency communication, and photovoltaic devices.

## Figures and Tables

**Table 1:** Molecular weight, thermal properties, and mechanical properties of PI 1~6.

PI	Mn/Da	Mw/Da	Mw/Mn	T_5_/°C ^1^	T_10_/°C ^2^	T_g_/°C ^3^	Y_800_/% ^4^	σt/MPa ^5^	εt/% ^6^	Et/MPa ^7^
PI 1	49.2 × 10^4^	57.5 × 10^4^	1.17	415.9	434.4	206.4	46.1	87.6	6.21	1.00 × 10^3^
PI 2	32.6 × 10^4^	39.7 × 10^4^	1.21	430.9	443.8	193.3	52.1	68.5	10.46	0.80 × 10^3^
PI 3	39.9 × 10^4^	46.4 × 10^4^	1.16	409.3	427.7	191.3	49.0	74.6	9.43	1.20 × 10^3^
PI 4	55.8 × 10^4^	59.9 × 10^4^	1.07	394.5	416.7	208.3	49.8	88.4	5.88	1.30 × 10^3^
PI 5	43.7 × 10^4^	51.5 × 10^4^	1.18	404.8	427.6	230.9	53.3	80.3	6.56	1.60 × 10^3^
PI 6	42.2 × 10^4^	48.6 × 10^4^	1.15	413.7	428.4	220.0	52.7	82.7	7.78	1.01 × 10^3^

^1^ 5% thermal weight loss temperature; ^2^ 10% thermal weight loss temperature; ^3^ glass transition temperature; ^4^ thermal weightlessness at 800 °C; ^5^ tensile strength; ^6^ elongation at break; ^7^ tensile modulus.

**Table 2:** Solubility of NFDN, NFDA, and PI 1~6.

	CHCI_3_	THF	EA	ET	TL	DMF	DMSO	DMAC
NFDN	+	+	+	+h	+h	+	+h	+
NFDA	+	+	+	+	+	+	+	+
PI 1	+	+	+	−	−	+	+	+
PI 2	+	+	+	−	+h	+	+	+
PI 3	+	+	+	−	−	+	+	+
PI 4	−	+	−	−	−	+	+	+
PI 5	−	−	±	+	−	+	+	+
PI 6	−	+h	−	−	+	−	+h	+

+: soluble at room temperature; −: insoluble; ±: slightly soluble at room temperature; +h: soluble after heating.

**Table 3:** Optical properties, calculated values for the frontier orbitals energies, interchain spacing, and contact angle of PI 1~6.

PI	Thickness/μm	T/% ^1^	λc/nm ^2^	E_HOMO_/eV	E_LUMO_/eV	ΔE/eV	CPS/Å ^3^	CA/^o 4^
PI 1	83	83.4	401	−5.42	−2.99	2.43	6.81	103.1
PI 2	72	86.7	376	−5.34	−2.42	2.92	6.37	98.1
PI 3	79	80.3	411	−5.32	−2.72	2.60	6.70	90.7
PI 4	85	56.2	471	−5.22	−3.64	1.58	4.78	109.4
PI 5	81	61.8	460	−5.15	−3.33	1.82	5.48	102.4
PI 6	78	85.7	377	−5.39	−3.34	2.05	5.89	91.3

^1^ Average transmittance in the 450–780 nm wavelength range; ^2^ cutoff wavelength; ^3^ intermolecular distance between polymer chains; ^4^ contact angle of the PI.

## Data Availability

The original contributions presented in this study are included in the article. Further inquiries can be directed to the corresponding author.
